# C3 Deficiency Leads to Increased Angiogenesis and Elevated Pro-Angiogenic Leukocyte Recruitment in Ischemic Muscle Tissue

**DOI:** 10.3390/ijms22115800

**Published:** 2021-05-28

**Authors:** Philipp Götz, Anna Braumandl, Matthias Kübler, Konda Kumaraswami, Hellen Ishikawa-Ankerhold, Manuel Lasch, Elisabeth Deindl

**Affiliations:** 1 Walter-Brendel-Centre of Experimental Medicine, University Hospital, Ludwig-Maximilians-Universität München, 81377 Munich, Germany; P.Goetz@med.uni-muenchen.de (P.G.); Anna.Braumandl@med.uni-muenchen.de (A.B.); Matthias.Kuebler@med.uni-muenchen.de (M.K.); Kumaraswami.Konda@med.uni-muenchen.de (K.K.); Hellen.Ishikawa-Ankerhold@med.uni-muenchen.de (H.I.-A.); manuel_lasch@gmx.de (M.L.); 2 Biomedical Center, Institute of Cardiovascular Physiology and Pathophysiology, Faculty of Medicine, Ludwig-Maximilians-Universität München, 82152 Planegg-Martinsried, Germany; 3 Department of Internal Medicine I, Faculty of Medicine, University Hospital, Ludwig-Maximilians-Universität München, 81377 Munich, Germany; 4 Department of Otorhinolaryngology, Head and Neck Surgery, University Hospital, Ludwig-Maximilians-Universität München, 81377 Munich, Germany

**Keywords:** angiogenesis, complement system, C3, leukocytes, macrophages, macrophage polarization, neutrophils, neutrophil extracellular traps, NETs, ischemia

## Abstract

The complement system is a potent inflammatory trigger, activator, and chemoattractant for leukocytes, which play a crucial role in promoting angiogenesis. However, little information is available about the influence of the complement system on angiogenesis in ischemic muscle tissue. To address this topic and analyze the impact of the complement system on angiogenesis, we induced muscle ischemia in complement factor C3 deficient (C3−/−) and wildtype control mice by femoral artery ligation (FAL). At 24 h and 7 days after FAL, we isolated the ischemic gastrocnemius muscles and investigated them by means of (immuno-)histological analyses. C3−/− mice showed elevated ischemic damage 7 days after FAL, as evidenced by H&E staining. In addition, angiogenesis was increased in C3−/− mice, as demonstrated by increased capillary/muscle fiber ratio and increased proliferating endothelial cells (CD31^+^/BrdU^+^). Moreover, our results showed that the total number of leukocytes (CD45^+^) was increased in C3−/− mice, which was based on an increased number of neutrophils (MPO^+^), neutrophil extracellular trap formation (MPO^+^/CitH3^+^), and macrophages (CD68^+^) displaying a shift toward an anti-inflammatory and pro-angiogenic M2-like polarized phenotype (CD68^+^/MRC1^+^). In summary, we show that the deficiency of complement factor C3 increased neutrophil and M2-like polarized macrophage accumulation in ischemic muscle tissue, contributing to angiogenesis.

## 1. Introduction

Vertebrates depend on the highly sophisticated structure of the circulatory system. The most filigree structure of this system, the capillary bed, is necessary for the exchange of gases, liquids, nutrients, signaling molecules, and cells between tissue and blood [1]. The development of new capillaries out of pre-existing vasculature, whether by sprouting or splitting, is denoted angiogenesis [2,3] and is substantial for a proper supply of nutrients and oxygen. Accordingly, this process plays an essential role in physiological and pathophysiological processes, such as cancer, inflammation, and wound healing [4]. In the context of vascular diseases, such as myocardial infarction or peripheral artery disease, capillary growth (angiogenesis) is essential for the removal of cell debris in areas of ischemic tissue damage caused by insufficient blood supply [5]. However, increased capillarity is not sufficient to restore blood supply. Only collateral artery growth (arteriogenesis) can compensate for the loss of an occluded artery [6].

In general, vascular endothelial growth factor A (VEGF-A) is one of the most potent angiogenic factors [1]. Hypoxia and hence ischemia is well described to increase VEGF-A levels in tissue [7,8]. However, also leukocytes, in particular neutrophils and monocytes, play an essential role in supplying VEGF-A [9,10,11,12]. Besides providing growth factors, macrophages and neutrophils play a crucial role in various steps of vessel formation, such as, for example, endothelial cell activation, matrix remodeling, and anastomosis of vessels [9,11,12]. Both types of leukocytes can enhance angiogenesis by providing pro-angiogenic factors and matrix metalloproteinases (MMPs), which are relevant for tissue remodeling [11,12]. To perform these tasks, leukocytes must be recruited, adhere, and migrate through the endothelial barrier, which is mediated by molecules such as selectins on the endothelial cell surface [13]. In particular, ICAM-1 was demonstrated to play a crucial role in the process of angiogenesis and arteriogenesis [14,15,16].

The complement system is a humoral part of the immune system, playing an important role in the innate and adaptive immune response. Discovered in the 19th century, the name was given by Paul Ehrlich to emphasize the complementary role in antibody-mediated lysis of pathogens [17]. Nowadays, the complement system is not merely referred to as the part of this immune response that eliminates bacteria by lysis but is also considered a crucial part of homeostasis and inflammation that is accordingly tightly regulated [18,19]. It is involved in the clearance of apoptotic cells, enhances coagulation, and acts, for example, as a co-stimulator in B cell activation [20].

The individual complement factors, mainly proenzymes, form a proteolytic cascade. This cascade can be activated through three different pathways, leading to the cleavage of the factor C3 to C3a and C3b [21]. C1q is the recognition particle of the classical pathway, normally interacting with pathogen-binding antibodies. Mannose-binding lectin (MBL) recognizes sugar structures of pathogens. When activated, both Cq1 and MBL lead to cleavage of C2 and C4, resulting in the formation of C3 convertase. In addition, the thioester bond of C3 undergoes spontaneous hydrolysis on a basal level known as tick-over. This alternative pathway (AP) is constitutively active and, in fact, the dominant activation pathway of the complement system under physiological conditions. To form the C3 convertase of the AP, factor B binds to activated C3 and is cleaved by the serine protease factor D forming the C3 convertase of the AP, C3bBb [21].

Factor C3 is the crucial link between activation pathways and effector mechanisms: C3b attaches itself to cell surfaces for opsonization and is required for C5 cleavage. While the resulting C5b is necessary to build the membrane attack complex (MAC), a cell lysis complex, C5a and C3a are anaphylatoxins, providing a strong chemotactic and inflammatory signal to leukocytes. Interestingly, there is evidence that C3a exhibits an inhibitory effect on neutrophils in contrast to its well-known pro-inflammatory effect on immune cells [22]. Moreover, the role of C3a and C5a is not limited to chemotaxis but extends to activation of leukocytes, enhancing the inflammatory response, e.g., by suppressing apoptosis and increasing antigen presentation [23,24]. While the liver is the systemic source for the complement, C3 and C5 in particular can be expressed by various other cells acting in an autocrine manner [24,25,26].

These functions of the complement system are not restricted to infectious diseases and do not always show beneficial effects but have been described to be involved in various sterile inflammatory diseases, too. For instance, complement deposition in the kidney is known to lead to glomerulopathy, in the eye to macular degeneration, and is also found in atherosclerotic plaques [25,27,28,29]. Moreover, C3 has been shown to promote reperfusion injury, e.g., in a diabetic model [30]. However, C3 seems to act anti-inflammatory as well. For example, the C3a receptor has been shown to play a protective role in atherosclerosis [31].

Concerning the function of C3 in angiogenesis, previous studies revealed controversial results: In a model for macular degeneration, C3 deficiency was shown to impair retinal vascularization [32], whereas increased angiogenesis was found in a mouse model of retinopathy of prematurity [33]. Interestingly, in vitro data have shown that endothelial cells (ECs) constitutively produce complement components, which can even be upregulated under pro-inflammatory and hypoxic conditions [26,34,35]. In addition, complement components can activate ECs to release chemotactic factors and to induce the expression of adhesion molecules, among them ICAM-1 [36].

Whether the complement system shows an impact on the process of angiogenesis in ischemic muscle tissue is still an enigma and was, therefore, the subject of the present study.

## 2. Results

To investigate the relevance of the complement system for angiogenesis in ischemic muscle tissue, we used a murine hindlimb model described by Limbourg et al. [37]. We induced tissue ischemia in gastrocnemius muscles of C3−/− and wildtype (WT) control mice by ligation of the right femoral artery (FAL) in adductor muscles. The left legs were sham-operated and served as internal negative controls. Gastrocnemius muscles were collected for (immuno-)histological analyses 24 h or 7 days after the surgery.

**Figure 1:**
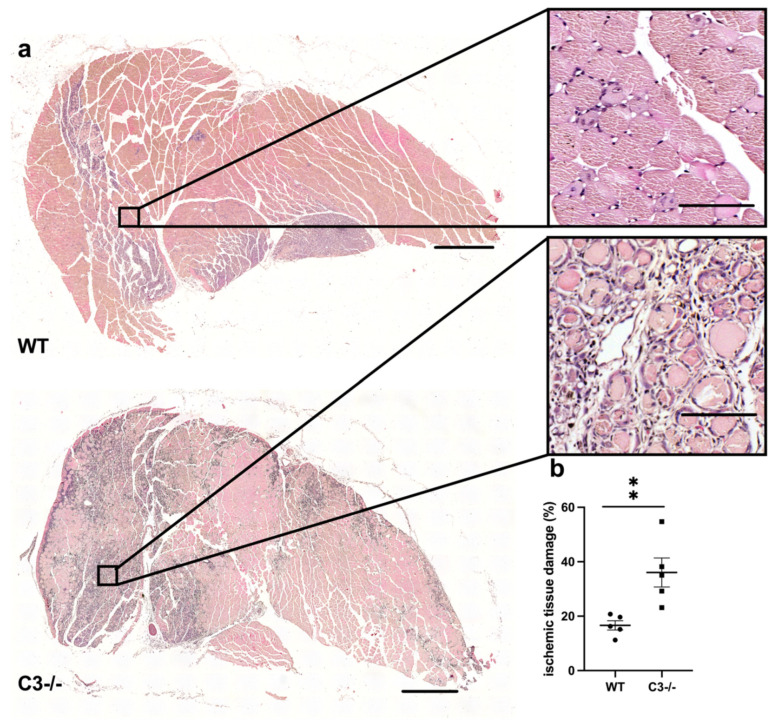
C3−/− mice show greater tissue damage. (**a**) Representative pictures of H&E stained gastrocnemius muscles of wildtype (WT) control mice (upper picture) and C3-deficient mice (lower picture) 7 days after FAL. Scale bars: 1000 µm (overview), 100 µm (detail). (**b**) Scatter plot shows the area of tissue damage (%) of C3−/− and WT mice 7 days after FAL. One complete sectional area was analyzed per mouse per group. Data shown are means ± SEM, *n* = 5 per group. ** *p* ≤ 0.01 (WT vs. C3−/−) by unpaired Student’s *t*-test.

Hematoxylin and eosin staining (H&E) confirmed ischemic damage of gastrocnemius muscles isolated from C3−/− and wildtype mice at day 7 day after FAL. However, compared to WT control mice, C3−/− mice showed significantly increased ischemic damage (Figure 1). Gastrocnemius isolated from sham-operated legs showed no signs of ischemic damage (for representative pictures, see Supplementary Materials, Figure S1).

To investigate whether C3 deficiency interferes with or promotes the process of angiogenesis in ischemic muscle tissue, we performed a CD31/ACTA2/BrdU/DAPI quadruple immunofluorescence staining on gastrocnemius muscles isolated 7 days after the surgical procedure. CD31 antibody was used as a marker for endothelial cells, and ACTA2 antibody as a marker for pericytes. DAPI was used to stain nuclei and BrdU as a marker for cell proliferation. Hence, only CD31^+^/ACTA2^−^/DAPI^+^ cells were counted as capillaries to exclude platelets that express CD31 but cannot be stained with DAPI. Muscle fibers were identified by their autofluorescence (see also Supplementary Materials, Figures S2 and S3). To quantify angiogenesis, we calculated the capillary to muscle fiber ratio.

**Figure 2:**
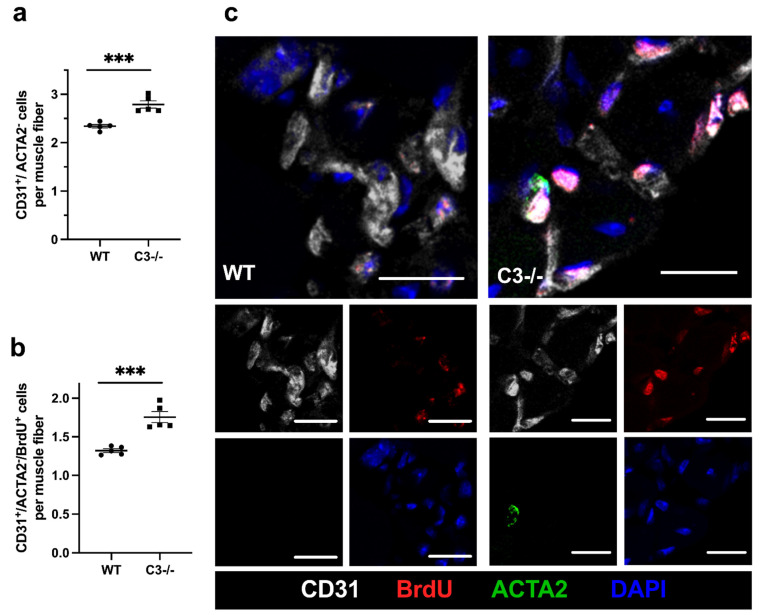
C3 deficiency enhances angiogenesis. The scatter plots show (**a**) endothelial cells (CD31^+^/ACTA2^−^) per muscle fiber as well as (**b**) proliferating endothelial cells (CD31^+^/ACTA2^−^/BrdU^+^) per muscle fiber in C3−/− and WT control mice in ischemic gastrocnemius muscles isolated 7 days after FAL. Data shown are means ± SEM, *n* = 5 per group, a defined ischemic area (1.5 mm^2^) of muscle tissue was analyzed per mouse. *** *p* ≤ 0.001 (WT vs. C3−/−) by unpaired Student‘s *t*-test. (**c**) Representative immunofluorescence pictures of analyzed ischemic gastrocnemius muscles of WT (left) and C3−/− mice (right) 7 days after FAL. Cells were labeled with antibodies targeting endothelial cells (CD31 (gray)), proliferating cells (BrdU (red)), pericytes (ACTA2 (green)) and with DAPI (blue) to label the nuclei. Scale bars: 20 µm.

Our results evidence that C3−/− mice showed a significantly increased capillary to muscle fiber ratio compared to WT control mice. (Figure 2). Counting only proliferating endothelial cells (CD31^+^/ACTA2^−^/BrdU^+^), we found again a significantly increased capillary to muscle fiber ratio in C3−/− compared to WT control mice. Investigations of non-ischemic gastrocnemius muscles isolated from sham-operated legs of C3−/− and WT control mice showed no differences in the capillary to muscle fiber ratio (data not shown; representative pictures are shown in the Supplementary Materials, Figure S3).

**Figure 3:**
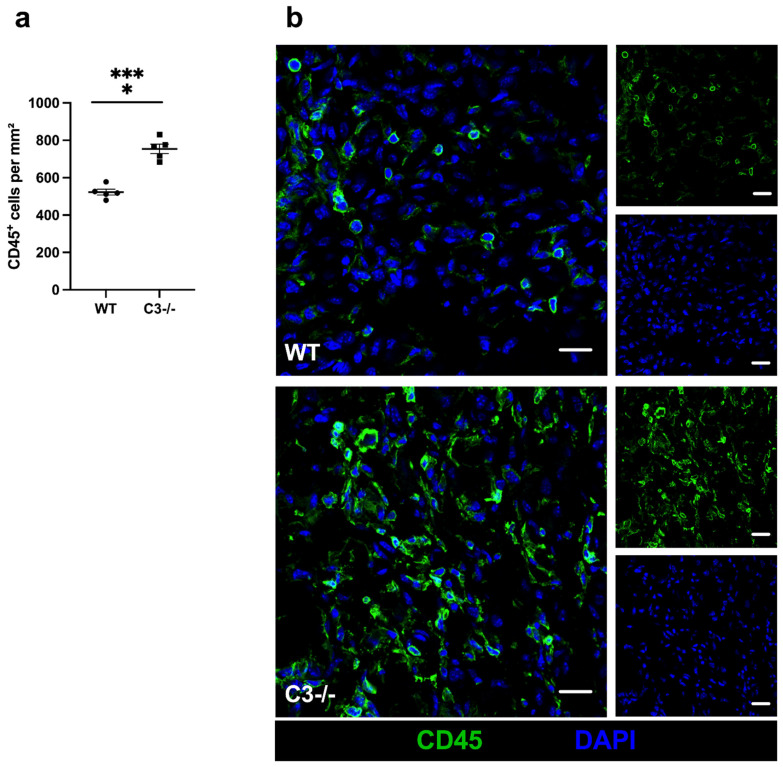
C3−/− mice show enhanced infiltration of leukocytes. (**a**) The scatter plot shows leukocytes (CD45^+^) per mm^2^ in ischemic gastrocnemius muscles isolated at day 7 after FAL. Data shown are means ± SEM, *n* = 5 per group, a defined ischemic area (1.5 mm^2^) of muscle tissue was analyzed per mouse. **** *p* < 0.0001 (WT vs. C3−/−) by unpaired Student‘s *t*-test. (**b**) Representative immunofluorescence pictures of analyzed gastrocnemius muscles of WT (upper picture) and C3−/− mice (lower picture) 7 days after FAL. Cells were labeled with antibodies targeting CD45 (green) and with DAPI (blue) to label nuclei. Scale bars: 20 µm.

Leukocytes are well described for their function in tissue regeneration and their contribution to angiogenesis. Investigating the total number of leukocytes using the pan-leukocyte marker CD45, we found significantly increased leukocytes in ischemic tissue of C3−/− mice compared to WT control mice 7 days after FAL (Figure 3).

**Figure 4:**
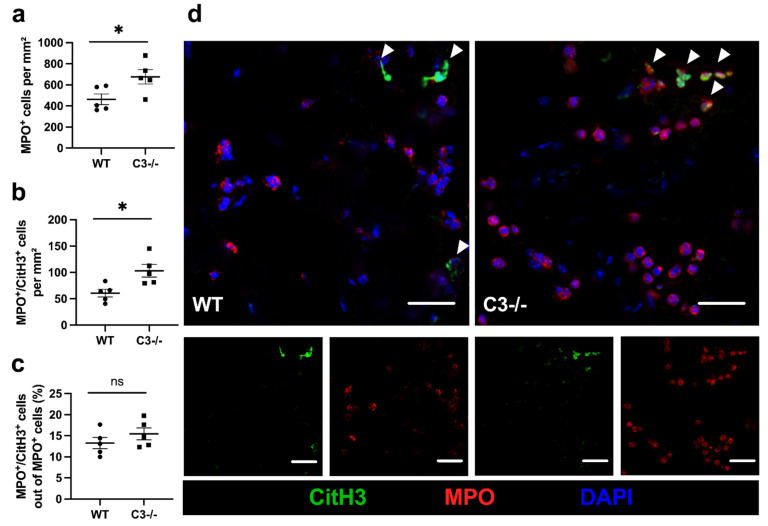
C3−/− mice show a higher number of neutrophils and NETs. Scatter plots show (**a**) neutrophils (MPO^+^) per mm^2^, (**b**) neutrophil extracellular traps (MPO^+^/CitH3^+^) per mm^2^ and (**c**) the percentage of NETs/neutrophils in ischemic gastrocnemius muscles isolated 24 h after FAL. Data shown are means ± SEM, *n* = 5 per group, a defined ischemic area (0.86 mm^2^) of muscle tissue was analyzed per mouse. * *p* < 0.05, ns ≥ 0.05 (WT vs. C3−/−) by unpaired Student‘s *t*-test. (**d**) Representative immunofluorescence pictures of analyzed ischemic gastrocnemius muscles of WT (left) and C3−/− mice (right). Cells were labeled with antibodies targeting MPO (red), CitH3 (green), and with DAPI (blue) to label nuclei. NETs (MPO^+^/CitH3^+^) are indicated by white arrowheads. Scale bars: 20 µm.

To obtain some further information about the contribution of the different leukocyte subsets, we performed a myeloperoxidase (MPO) stain to investigate the number of recruited neutrophils at 24 h after FAL. Moreover, we stained against citrullinated histone H3 (Cit-H3) to detect neutrophil extracellular traps (NETs). Our results showed that the total number of neutrophils as well as the number of NETs was significantly increased in C3−/− mice compared to WT control mice, while the NETs to neutrophil ratio remained unchanged (Figure 4).

**Figure 5:**
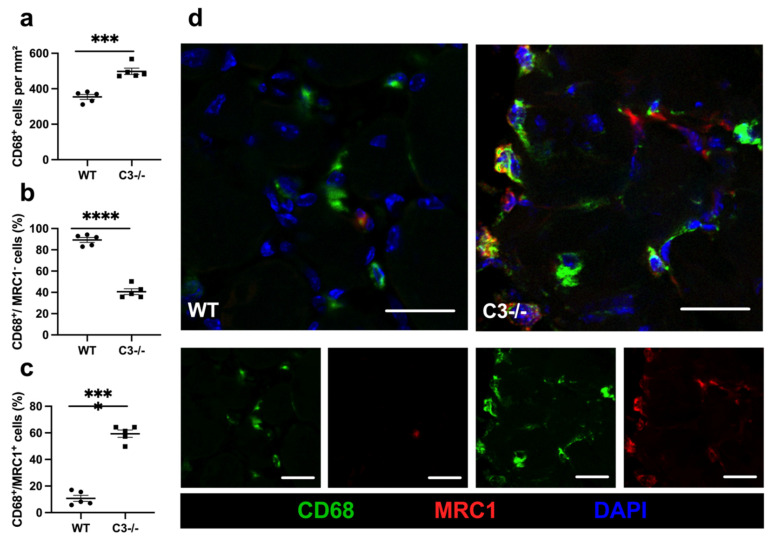
C3 deficiency goes along with increased macrophage recruitment and M2-like polarization on day 7 after surgery. Scatter plots show (**a**) macrophages (CD68^+^) per mm^2^, (**b**) the percentage of M1-like polarized (CD68^+^/MRC1^−^), and (**c**) the percentage of M2-like polarized macrophages (CD68^+^/MRC1^+^) in ischemic gastrocnemius muscles 7 days after FAL. Data are shown as means ± SEM, *n* = 5 per group. *** *p* ≤ 0.001, **** *p* < 0.0001 (WT vs. C3−/−) by unpaired Student‘s *t*-test. (**d**) Representative immunofluorescence pictures of analyzed ischemic gastrocnemius muscles of WT (left) and C3−/− mice (right) 7 days after FAL. Cells were labeled with antibodies targeting MRC1 (red), CD68 (green), and with DAPI (blue) to label the nuclei. Scale bars: 20 µm.

To analyze the contribution of macrophages on angiogenesis in ischemic muscle tissue, we performed a macrophage staining using CD68 to label the total number of macrophages and added an antibody against mannose receptor C-type 1 (MRC1) to distinguish between M1-like (MRC1^−^) and M2-like (MRC1^+^) polarized macrophages. On day 7 after the surgical intervention, we found a significant increase in the total number of macrophages, with a predominance of M2-like polarized macrophages and a significant decrease in M1-like polarized macrophages in C3−/− mice compared to control mice (Figure 5). In gastrocnemius muscles isolated from legs of sham-operated mice, only a little number of leukocytes in general (CD45^+^ cells) as well as neutrophils (MPO^+^ cells), and macrophages (CD68^+^ cells) in particular were seen. Moreover, there was no difference in number between WT and C3−/− mice (data not shown; for representative pictures, see Supplementary Materials Figure S4). NETs were not seen at all in gastrocnemius muscles isolated from sham-operated legs (for representative pictures, see Supplementary Materials Figure S4).

## 3. Discussion

In the present study, we analyzed the influence of C3-deficiency on angiogenesis in ischemic muscle tissue. Our findings show that the absence of C3 was associated with increased ischemic tissue damage and enhanced angiogenesis. Increased immune cell infiltration (CD45^+^ cells), seen in C3−/− mice, was based on increased infiltration of neutrophils (MPO^+^) as well as macrophages (CD68^+^) and NET formation (MPO^+^/CitH3^+^). In contrast to WT controls, in C3-deficient mice, macrophages were predominantly polarized to a pro-angiogenic M2-like (CD68^+^/MRC1^+^) phenotype. Together, our data correspond to the current state of knowledge concerning the participation of leukocytes in angiogenesis and suggest that improved angiogenesis in ischemic muscle tissue of C3−/− mice is at least in part mediated by the increased number of pro-angiogenic leukocytes.

Compared to WT control mice, our results showed increased tissue damage in C3−/− mice. In our model, FAL results in arteriogenesis in the upper leg and, due to reduced perfusion, to ischemic tissue damage in the lower leg, i.e., in the gastrocnemius muscle [37]. Chillo et al. demonstrated that improved arteriogenesis leads to reduced tissue damage in the lower leg [38]. Accordingly, impaired arteriogenesis results in increased distal ischemic tissue damage. Whether collateral artery growth in the upper leg of C3-deficient mice is in fact impaired and thus causative for the observed increased ischemic tissue damage of the lower leg remains to be investigated. We did not observe any difference in the number of preexistent and growing collaterals between WT and C3−/− mice (data not shown). Both processes, arteriogenesis and angiogenesis, require the recruitment of leukocytes to provide growth factors, cytokines, and MMPs, enhancing cell proliferation, further leukocyte recruitment, and matrix remodeling consequently. While angiogenesis is based mainly on the proliferation of endothelial cells (ECs), arteriogenesis requires the proliferation of endothelial and smooth muscle cells (SMCs) [39]. In the absence of C3, EC proliferation is not disturbed but even increased in angiogenesis, as we have demonstrated here. This might indicate that EC proliferation is not affected in arteriogenesis either. Though, this is speculative. In addition, it remains to be investigated whether SMC proliferation is hampered in arteriogenesis resulting in a decreased diameter of growing collaterals. Moreover, any translation of our findings to the context of arteriogenesis is limited, as mechanisms of arteriogenesis and angiogenesis differ [39]. While ischemia is the driving force for angiogenesis, arteriogenesis is promoted by shear stress [40,41]. Ultimately, the role of the complement system in arteriogenesis remains to be elucidated in separate in-depth studies. However, our data are of particular importance when seen in the context of ischemia/reperfusion injury (I/R injury) since it has been discussed that inhibition of the complement system may present an appropriate therapeutic approach to minimize I/R injury [42,43]. Indeed, it has been shown that deficiency of components of the complement system results in reduced I/R injury; however, other studies have demonstrated the complete opposite and have shown that activation of the complement system ameliorated I/R injury [22,44].

Since strong connections between innate immune cells and the complement system are well known, we analyzed leukocyte infiltration in the ischemic muscle tissue of our hind limb model in C3-deficient mice. C3 deficiency led to higher infiltration of leukocytes, particularly to increased numbers of macrophages and neutrophils.

In damaged tissue, infiltrating leukocytes participate in tissue remodeling. Local inflammation leads to a leakier endothelium, facilitating the formation of new blood vessels and the transmigration of leukocytes [12]. Leukocytes remove cell debris and enhance further leukocyte recruitment by boosting local inflammation. Especially neutrophils and macrophages release pro-angiogenic growth factors, including VEGF [9], and are further relevant for the remodeling of extracellular matrix by releasing proteases, such as matrix metalloproteinase 9 (MMP9) [11,12,45,46]. Since anaphylatoxins C3a and C5a are chemoattractants for several leukocyte subpopulations [18], the absence of C3 could results in less leukocyte infiltration, as it has been described earlier in the context of angiogenesis [25]. However, our data demonstrate the opposite effect, and C3 seems to play an inhibitory function for leukocyte recruitment to the ischemic tissue. One explanation may be that the lack of the chemoattractants C3a and C5a are compensated or even over-compensated in C3-deficient mice by the release of other chemoattractant signals, such as damage-associated molecular patterns (DAMPs), which are liberated upon tissue damage. DAMPs act as strong chemoattractants and induce the release of chemokines and lipid mediators from ischemic tissues [47,48]. In addition, recruited leukocytes may reinforce this signal in a manner of a positive feedback loop and provide further growth factors relevant for vascular cell proliferation.

The complement system is considered to be a complex inert immune surveillance system, playing an important role in several processes of inflammation [18]. As inflammation is a major component in vascular diseases and repair [2,49,50,51], the contribution of the complement system has been (and still is) thoroughly investigated. Thereby, the generation of the anaphylatoxins (C3a, C5a) and the opsonization via C3b and MAC formation are focused topics. C3-deficient mice are commonly used to investigate the contribution of the complement system in processes of vascularization. However, there is evidence that C3 can be circumvented and that C5 might be cleaved in the absence of C3 [52]. Nevertheless, it is known that the complement activation and C3, as the connector between the activation pathways and effector mechanisms, is involved in cardiovascular diseases such as arteriosclerosis [53,54,55,56].

In the early phase of inflammation and angiogenesis, neutrophils play a crucial role as they are recruited to damaged tissues in response to inflammatory signals, most likely DAMPs. Neutrophils, as highly potent phagocytes, participate in tissue repair and cell debris removal. More importantly, they efficiently provide pro-angiogenic growth factors and are able to interact with endothelial cells to induce VEGF production and enhance angiogenesis [45]. The relevance of neutrophils in initiating angiogenesis was pointed out by the fact that neutropenic mice failed to revascularize transplanted pancreatic islets and that neutrophil depletion led to a strong reduction in angiogenesis [45,57]. In our study, we found an increased number of neutrophils, which is in accordance with previous findings demonstrating that neutrophil mobilization, migration, and degranulation are inhibited by C3a [18,22]. Together with the above-mentioned and in the literature described functions of neutrophils, our data suggest that the increased number of neutrophils contributed to the improved angiogenesis found in ischemic muscle tissue of C3−/− mice. However, neutrophils may also have contributed to increased ischemic tissue damage as they are known to release toxic effectors such as reactive oxygen species (ROS) [58]. The same may apply to the NETs in our study.

In our investigations, we found an increased number of NETs in ischemic tissue of C3-deficient mice—although the number of NETs in relation to the number of neutrophils was not changed. This indicates that C3 deficiency did not influence NET formation in ischemic muscle tissue. These data are in contrast to previous findings showing that C3 deficiency is hampered in infection-induced NETosis [59]. Together, these results suggest that NET formation as a function of micro-environmental conditions is either dependent or not dependent on the complement factor C3. We propose that under conditions of ischemia and sterile inflammation, such as in our model of FAL-induced muscle ischemia, high levels of DAMPs (which are known to be potent activators of NET formation [60]) are necessary and sufficient to induce NETosis. Anyhow, it has been demonstrated that NET formation is not only associated but can also be causative for tissue damage [61,62]. On the other hand, NETs can also promote angiogenesis and vascular regeneration [63,64]. Accordingly, in the case of ischemic tissue damage, NETs may promote tissue damage; however, they may also contribute to improved angiogenesis in C3-deficient mice.

Our data demonstrated that in ischemic muscle tissue, the capillary to muscle fiber ratio and rate of proliferating endothelial cells is increased in C3−/− mice compared to WT mice. As we used CD31 as an endothelial marker, it should be mentioned that CD31 expression is not restricted to endothelial cells but can be expressed on platelets and leukocytes, too [65,66]. To exclude platelets, only CD31 signals that colocalized with nucleic signals (DAPI) were counted. A CD45/CD31 co-staining barely showed colocalizations of CD45 and CD31 (data not shown). Therefore, we used markers to exclude platelets and pericytes but no marker to exclude leukocytes for the quantification of capillaries. In the past, several studies have already been performed on the crosslink between C3 deficiency and angiogenesis: In a model of age-related macular degeneration (AMD), inducing choroidal neovascularization (CNV) by laser photocoagulation, Bora et al. showed that C3-deficient mice were protected against CNV [32]. As the depletion of C6, necessary for MAC formation, showed similar results, they proposed that the deposition of MAC on retinal pigment endothelium (RPE) and/or on the choroid induces growth factor release. However, this does not seem to play an important role in ischemia-induced angiogenesis: Using a hypoxia-induced model of retinal vascularization, Langer et al. demonstrated that C3−/− mice show increased angiogenesis compared to their controls [33]. In particular, their results suggested that anaphylatoxins keep macrophages in inflammatory M1 polarization suppressing angiogenesis by releasing anti-angiogenic factors such as soluble VEGF receptor-1, which lowers the bioavailability of VEGF. Interestingly, this group already hypothesized that their findings might not be only restricted to the retinal. In our study on ischemic muscle tissue, we found in C3-deficient mice a reduced number of M1-like polarized macrophages but an increased number of M2-like polarized macrophages, which are well described for their pro-angiogenic function [67,68].

In general, the presence of macrophages, which are a diverse group of cells with high plasticity, has a substantial impact on angiogenesis at the side of ischemic tissue [12]. Stimuli-dependent, macrophages polarize to predominantly pro-inflammatory (M1-like (Cd68+/MRC1^−^)) or anti-inflammatory (M2-like (Cd68^+^/MRC1^+^)) phenotypes, although M1- and M2-like polarization represent two extremes of a wide polarization spectrum [69]. In the early phase of inflammation, macrophages show a pro-inflammatory phenotype and are involved in removing cell debris, releasing growth factors, and remodeling the extracellular matrix [69,70]. M1-like macrophages are important at the beginning of the process of angiogenesis by delivering pro-angiogenic factors such as TNFα and VEGF, and in zebrafish, it has been shown that a lack of pro-inflammatory macrophages counteracts angiogenesis [71]. However, the impact of M1-like macrophages on angiogenesis is limited: M2-like macrophages are required for matrix remodeling, as, e.g., matrix metalloproteinase 9, a potent enzyme for matrix remodeling, is complexed and therefore inactive in M1-like macrophages [72]. Thus, M2-like macrophages remain to be the classical pro-angiogenic phenotype being associated with angiogenesis [68,73]. Interestingly, the complement component C3a has previously been demonstrated to induce pro-inflammatory macrophage polarization [74,75]. This finding fits very nicely with our results on the reduced number of pro-inflammatory M1-like polarized macrophages and increased number of M2-like polarized pro-angiogenic macrophages and emphasizes our data on improved angiogenesis in ischemic muscle tissue of C3-deficient mice.

However, we do not want to omit the data from Nozaki et al., who showed in an accelerated model of age-related macular degeneration that genetic ablation of C5aR or C3aR resulted in a reduced recruitment of leukocytes and reduced VEGF levels along with reduced choroidal neovascularization [25]. Our immunohistological analyses, however, evidenced a higher infiltration of macrophages at the side of tissue ischemia as well as a notable increase in the percentage of regenerative M2-like polarized macrophages in C3−/− mice, characterized by MRC1 expression. WT and C3−/− mice underwent the same surgical procedure. Nevertheless, significant differences in leukocyte infiltration and angiogenesis occurred by a direct comparison of the damaged area of the muscle tissue. Our data reveal that C3 deficiency leads to an elevated infiltration of leukocytes and a pro-angiogenic shift in the leukocyte subset of macrophages on the one hand and increased angiogenesis on the other hand. Although not we did not investigate whether the increased number of leukocytes seen in our animal model of hindlimb ischemia is responsible for the observed improved angiogenesis, our data are in accordance with the current literature concerning the participation of leukocytes in angiogenesis. In particular, our findings of an increased M2-like polarized macrophage infiltration and ameliorated angiogenesis in C3−/− mice go along with the results of Langer et al. in their model of hypoxia-induced retinal vascularization [33]. Their conclusion that C5a, which binds to macrophage’s C5a receptor, is crucial to keep macrophages in an M1-like polarization showing an anti-angiogenic effect is likely to apply also for our findings. In accordance with their data, the predominance of M2-like polarized macrophages in C3−/− mice in our model may be the result of a missing activation of the C5a/C5aR axis. Together, our findings indicate that blocking the complement factor C3 is a potential tool to improve angiogenesis in ischemic muscle tissue by inducing M2-like macrophage polarization.

In summary, we show that C3 deficiency increased capillarity in ischemic muscle tissue. Increased accumulation of leukocytes, in particular neutrophils, pro-angiogenic M2-like polarized macrophages, and NETs are likely to constitute the basis for the observed improvement of angiogenesis in mice deficient for the complement factor C3.

## 4. Materials and Methods

### 4.1. Animals and Experimental Procedures

Experiments and animal care were permitted by the Bavarian Animal Care and Use Committee (ethical approval code: ROB-55.2Vet-2532.Vet_02-17-99) and performed in strict accordance with German and NIH animal legislation guidelines. Experiments included C3-deficient mice (C3^tm1Crr^, JAX stock #029661, The Jackson Laboratory, referred here as C3−/−), bred at the institute, and wildtype (WT), C57BL/6J mice, provided by Charles River Laboratory (Sulzfeld, Germany).

8–10 weeks old mice underwent surgical procedure: anesthetized with a combination of fentanyl (0.05 mg/kg, CuraMED Pharma, Karlsruhe, Germany), midazolam (5.0 mg/kg, Ratiopharm GmbH, Ulm, Germany), and medetomidine (0.5 mg/kg, Pfister Pharma, Berlin, Germany), the right femoral artery was ligated to set an ischemic stimulus leading to angiogenesis in the gastrocnemius muscle, while the left leg was sham-operated as previously described [37]. BrdU was administered daily i.p. as a proliferation marker (1.25 mg, Sigma-Aldrich, St. Louis, MO, USA), dissolved in 100 µL phosphate-buffered saline (PBS, 148 mM Na^+^, 1.8 mM K^+^, pH 7.2), starting directly after FAL. Tissue was collected 24 h or 7 days after FAL (every timepoint and group *n* = 5): After the mice were sacrificed, they were first perfused with adenosine buffer (1% adenosine (Sigma-Aldrich), 5% bovine serum albumin (BSA, Sigma-Aldrich), dissolved in PBS) and afterward with 3% paraformaldehyde (PFA, Merck, Darmstadt, Germany) for cryopreservation or 4% PFA for paraffin embedding. Finally, gastrocnemius muscles were removed and stored.

### 4.2. Histological and Immunofluorescence Analysis

For immunohistology, staining was performed with 8 µm thick cryosections of the gastrocnemius muscle. Tissue of day 7 after FAL was used to stain for endothelial cells, leukocytes, and macrophages. Whereas neutrophil infiltration and NETs were analyzed in tissue samples isolated 24 h after FAL. Sections were incubated with 1N HCl for 30 min at 37 °C to bare BrdU in nuclei, blocked with 10% goat serum, dissolved in 4% BSA PBS/0.1% Tween-20 (Tween 20, AppliChem GmbH, Darmstadt, Germany) for 1 h at room temperature (RT) and then incubated with anti-BrdU antibody (1:50, Abcam, ab6326) at 4 °C overnight. Goat anti-rat Alexa Flour^®^ 546 antibody (1:100, Thermo Fischer, A-11081) was used as a secondary antibody, followed by an Alexa Flour^®^ 647 anti-mouse CD31 (1:50, BioLegend, 102516) and anti-ACTA2-Alexa Flour^®^ 488 (1:400, Sigma-Aldrich, F3777), to stain endothelial cells (CD31^+^/ACTA2^−^) and CD31^+^-pericytes (CD31^+^/ACTA2^+^). Leukocytes were labeled using an anti-CD45-Alexa Fluor^®^ 488 antibody (1:100, Thermo Fisher, 11-0451-85) at 4 °C overnight.

Macrophages were labeled with anti-CD68-Alexa Flour^®^ 488 (1:200, Abcam, ab201844) and anti-MRC1 antibody (1:200, Abcam, ab64693) at 4 °C overnight, followed by the secondary antibody donkey anti-rabbit-Alexa Flour^®^ 546 (1:200, Thermo Fisher, A10040). To analyze NETs at day 1, samples were stained for myeloperoxidase (MPO) and citrullinated histone H3 (Cit-H3). Samples were incubated with a polyclonal goat anti-MPO antibody (1:20, R&D Systems, AF3667) and a polyclonal rabbit anti-Cit-H3 antibody (1:100, Abcam, ab5103) at 4 °C overnight, followed by secondary antibodies donkey anti-goat-Alexa Fluor^®^ 594 antibody (1:100, Thermo Fisher, A-11058) and donkey anti-rabbit-Alexa Fluor^®^ 488 antibody (1:200, Thermo Fisher, A-21206) for 1 h at RT. Additionally, all tissues were incubated with DAPI (1:1000, Thermo Fisher, 62248) for 10 min at RT to label nucleic DNA. For mounting, Dako mounting medium was used (Dako, Agilent, Santa Clara, CA, USA). The mounted tissue of C3−/− and control mice (*n* = 5 per group and timepoint) was analyzed by counting cells of an ischemic area of 1.5 mm^2^ (CD45^+^ cells, CD68^+^ cells) or 0.86 mm^2^ for neutrophils. To investigate angiogenesis, the capillary to muscle fiber ratio was calculated as previously described [76]. CD31^+^/ACTA2^−^/DAPI^+^ cells were counted as capillaries. Muscle fibers were identified by their auto-fluorescent signal, and the capillary to muscle fibers ratio was used as angiogenesis index. Images for cell counting were taken with a 20 × objective either with a confocal laser scanning microscope LSM 880 by Carl Zeiss AG (CD68/MRC1/DAPI staining, MPO/CitH3/DAPI staining) or an epifluorescence microscope DM6 B by Leica (CD31/ACTA2/BrdU/DAPI staining, CD45/DAPI staining). Confocal images of (425 × 425 µm) per muscle were analyzed in case of CD68/MRC1 quantification, 5 confocal pictures (425 × 425 µm) per muscle were analyzed in case of MPO/CitH3 quantification. For CD31/BrdU/ACTA2 staining and CD45 staining 5 epifluorescence pictures (630 × 475 µm) were analyzed. ZEN blue software (Carl Zeiss AG) and the open-source program ImageJ were used for counting analyses.

For the H&E staining, 5 µm thick paraffin-embedded gastrocnemius muscle sections (*n* = 5 per group) were used, and the total necrotic area (%) of the whole tissue slices was quantified using ImageJ.

### 4.3. Statistical Analyses

Our analyses were carried out with GraphPad Prism 8 (GraphPad Software, LA Jolla, CA, USA). Data are mean values ± standard error of the mean (SEM). We consider the findings to be statistically significant at *p* < 0.05.

## Data Availability

The data presented in this study is available on request from the first author.

## Supplementary Materials

The following are available online at https://www.mdpi.com/article/10.3390/ijms22115800/s1.
