# Supplementary material for: C3 Deficiency Leads to Increased Angiogenesis and Elevated Pro-Angiogenic Leukocyte Recruitment in Ischemic Muscle Tissue
Source: Int J Mol Sci. 2021 May 28;22(11):5800. doi: 10.3390/ijms22115800 (PMC8198161; doi:10.3390/ijms22115800)
Supplement: Supplementary file 1 [file ijms-22-05800-s001.zip › Supplementary material/ijms-1203730 supplementary material.pdf]

International Journal of *Molecular Sciences*

MDPI

Article

C3 Deficiency Leads to Increased Angiogenesis and Elevated Pro-Angiogenic Leukocyte Recruitment in Ischemic Muscle Tissue

Philipp Götz [1,2], Anna Braumandl [1,2], Matthias Kübler [1,2], Konda Kumaraswami [1,2], Hellen Ishikawa-Ankerhold [1,3,†], Manuel Lasch [1,2,4,†] and Elisabeth Deindl [1,2,*,†]

[1] Walter-Brendel-Centre of Experimental Medicine, University Hospital, Ludwig-Maximilians-Universität München, 81377 Munich, Germany; P.Goetz@med.uni-muenchen.de (P.G.); Anna.Braumandl@med.uni-muenchen.de (A.B.); Matthias.Kuebler@med.uni-muenchen.de (M.K.); Kumaraswami.Konda@med.uni-muenchen.de (K.K.); Hellen.Ishikawa-Ankerhold@med.uni-muenchen.de (H.I.-A.); manuel_lasch@gmx.de (M.L.)

[2] Biomedical Center, Institute of Cardiovascular Physiology and Pathophysiology, Faculty of Medicine, Ludwig-Maximilians-Universität München, 82152 Planegg-Martinsried, Germany

[3] Department of Internal Medicine I, Faculty of Medicine, University Hospital, Ludwig-Maximilians-Universität München, 81377 Munich, Germany

[4] Department of Otorhinolaryngology, Head and Neck Surgery, University Hospital, Ludwig-Maximilians-Universität München, 81377 Munich, Germany

* Correspondence: Elisabeth.Deindl@med.uni-muenchen.de; Tel.: +49-(0)-89-2180-76504

† These authors contributed equally to this work.

Citation: Götz, P.; Braumandl, A.; Kübler, M.; Kumaraswami, K.; Ishikawa-Ankerhold, H.; Lasch, M.; Deindl, E. C3 Deficiency Leads to Increased Angiogenesis and Elevated Pro-Angiogenic Leukocyte Recruitment in Ischemic Muscle Tissue. *Int. J. Mol. Sci.* **2021**, *22*, 5800. https://doi.org/10.3390/ijms22115800

Academic Editor: Javier Conde Aranda and Morena Scotece

Received: 15 April 2021
Accepted: 27 May 2021
Published: 28 May 2021

Publisher's Note: MDPI stays neutral with regard to jurisdictional claims in published maps and institutional affiliations.

Copyright: © 2021 by the authors. Submitted for possible open access publication under the terms and conditions of the Creative Commons Attribution (CC BY) license (http://creativecommons.org/licenses/by/4.0/).

Supplementary Material

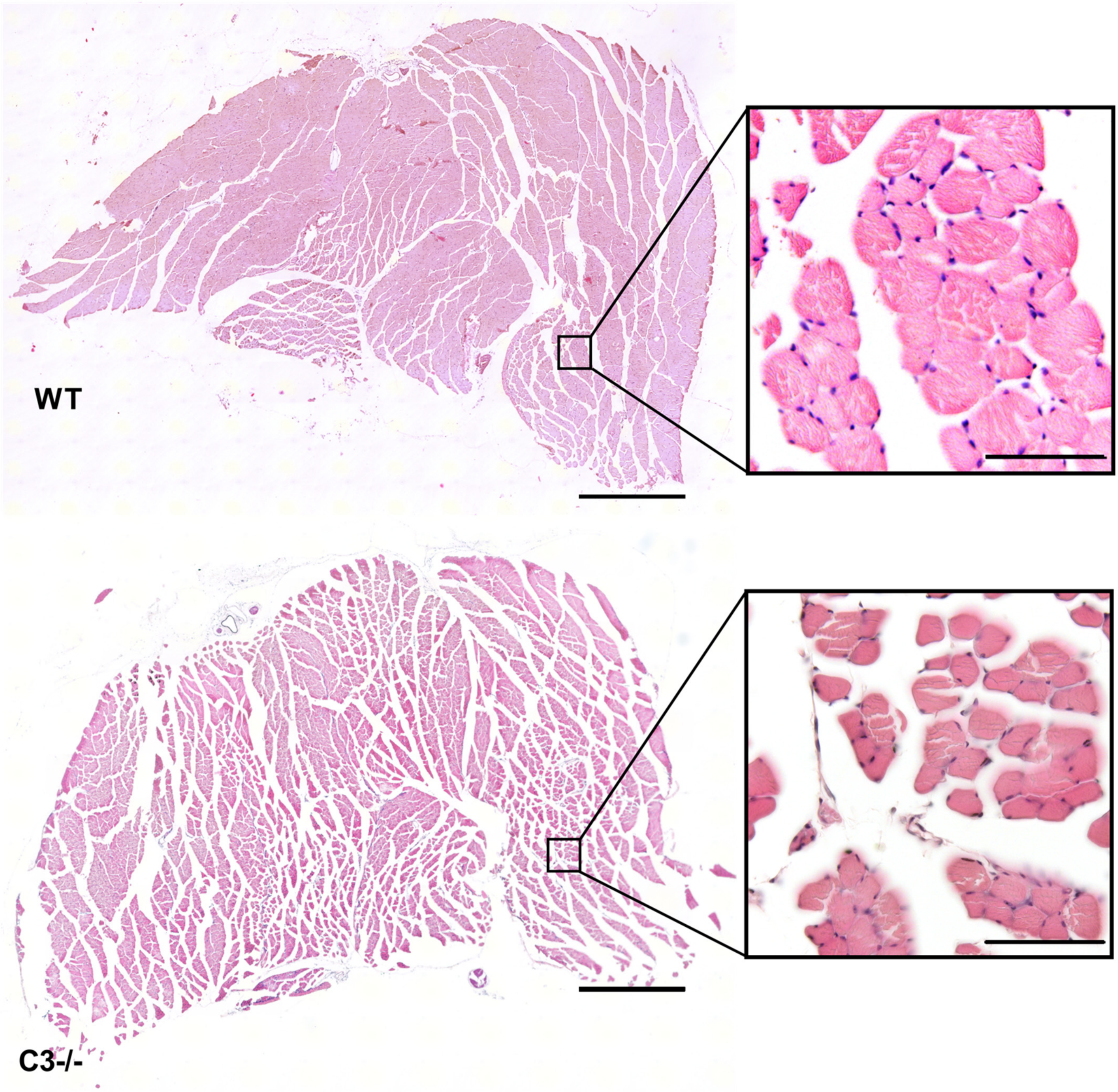

Figure S1. Tissue from sham-operated legs (non-ischemic) show no signs of ischemic damage. Representative pictures of H&E stained gastrocnemius muscles isolated from sham-operated legs of wildtype (WT) mice (upper picture) and C3−/− mice (lower picture) 7 days after FAL. Scale bars: 1000 µm (overview), 50 µm (detail).

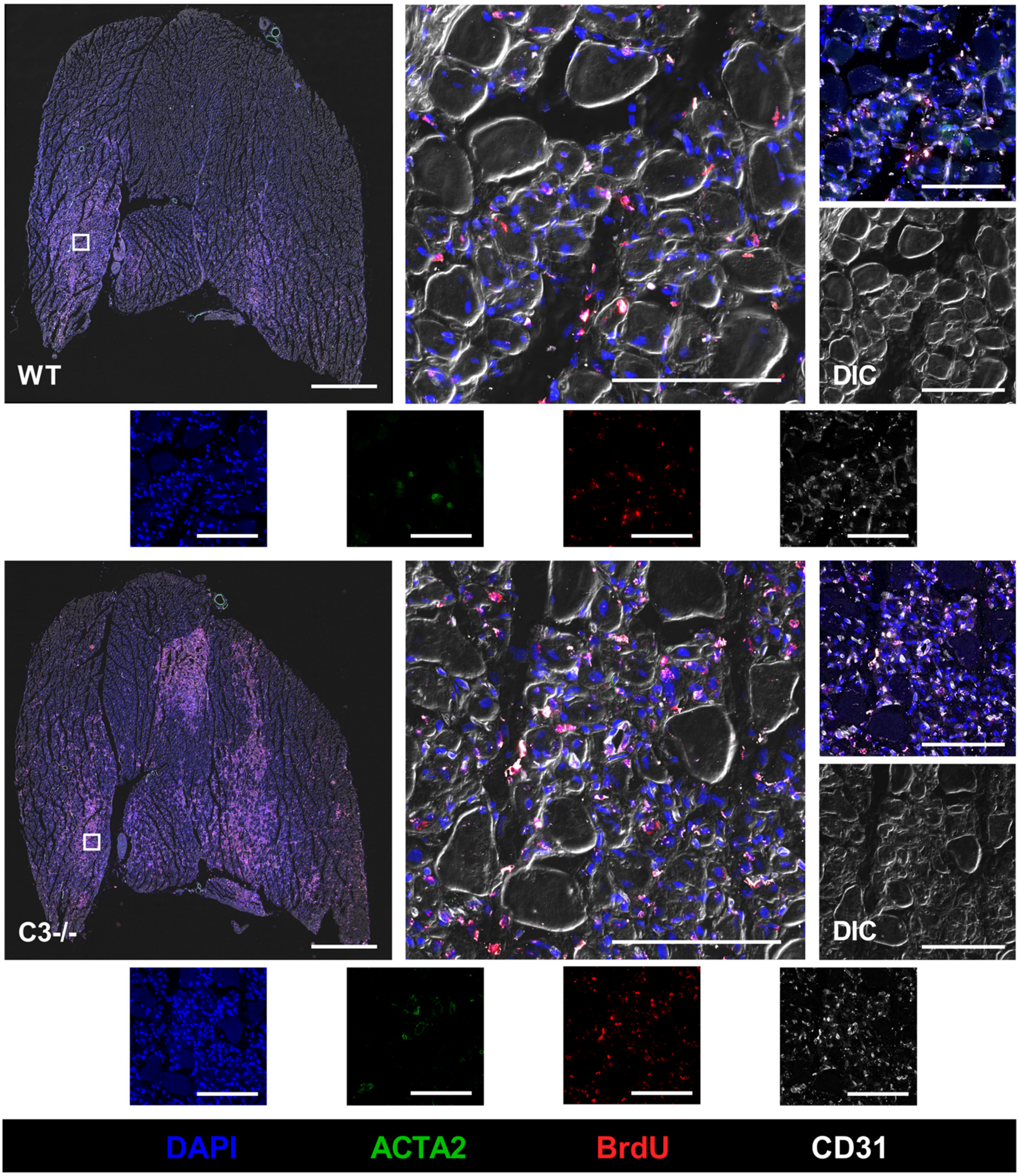

Figure S2. Ischemic gastrocnemius muscles of C3−/− mice show increased angiogenesis. Representative pictures of ischemic gastrocnemius muscles of WT (upper pictures) and C3−/− mice (lower pictures) 7 days after FAL. Immunofluorescence pictures were combined with brightfield images taken with polarized light (differential interference contrast, DIC) to show the muscle fiber structures. ***Left:*** Several pictures were stitched to display the whole sectional area of approx. 20 mm^2. White square indicates detail. ***Middle***: Detail is displayed as zoomed images of IF+DIC. ***Right:*** Individual IF and DIC pictures are shown, ***below*** IF single channels. Cells were labeled with antibodies targeting endothelial cells (CD31$^+$ (grey)), proliferating cells (BrdU$^+$ (red)), pericytes (ACTA2$^+$ (green)), and stained with DAPI (blue) to label the nuclei. Scale bars: 1000µm (overview), 100µm (detail).

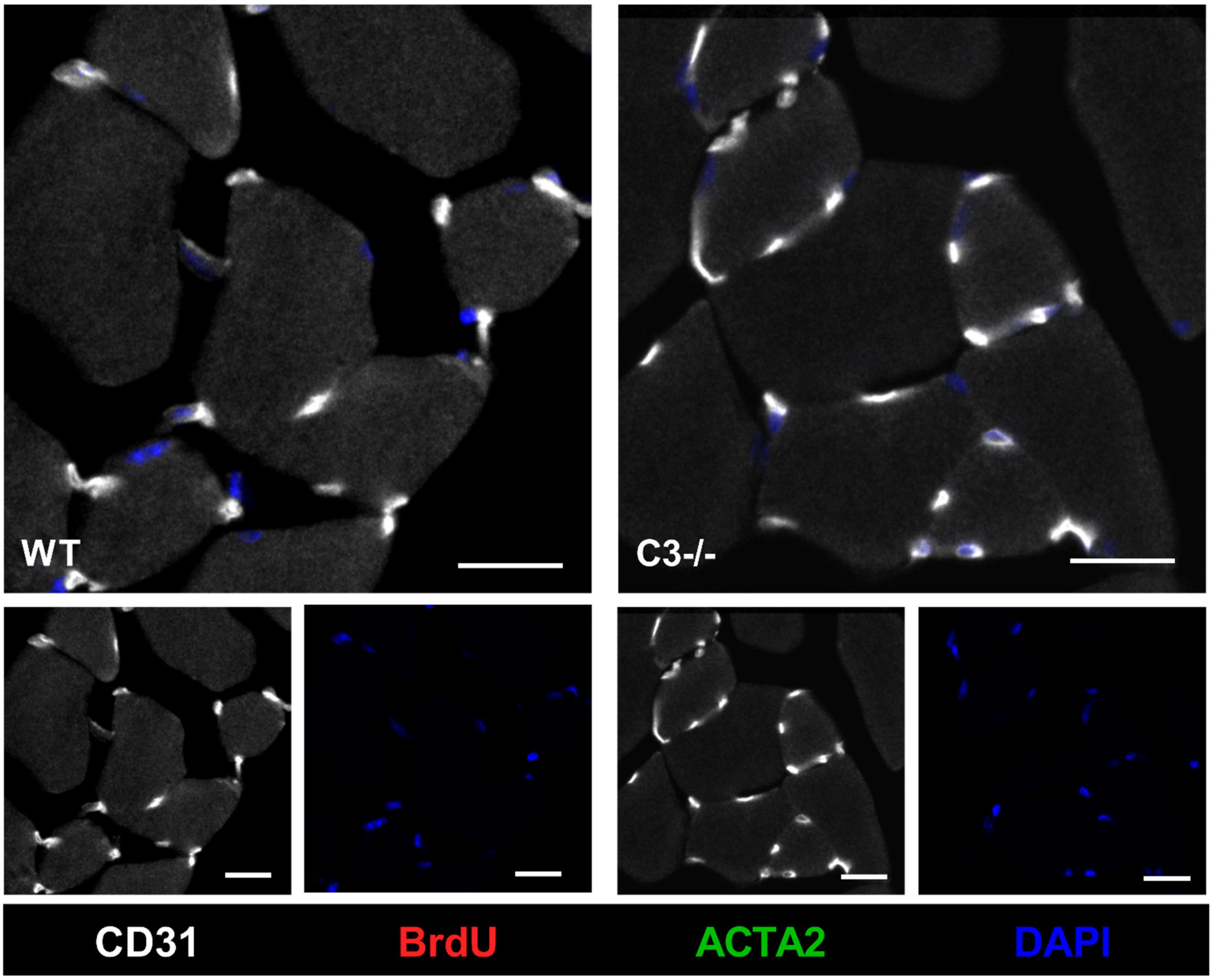

Figure S3. C3−/− and WT mice show a comparable number of endothelial cells per muscle fiber. Immunofluorescence pictures of gastrocnemius muscles isolated from sham-operated (non-ischemic) legs of WT (left) and C3−/− mice (right) 7 days after FAL. No proliferation (BrdU$^+$ cells) is seen. Cells were labeled with antibodies targeting endothelial cells (CD31$^+$ (grey)), proliferating cells (BrdU$^+$ (red)), pericytes (ACTA2$^+$ (green)), and stained with DAPI (blue) to label the nuclei. Only single channels of CD31 and DAPI are displayed as the channels for BrdU and ACTA showed no signal. Scale bars: 20 µm.

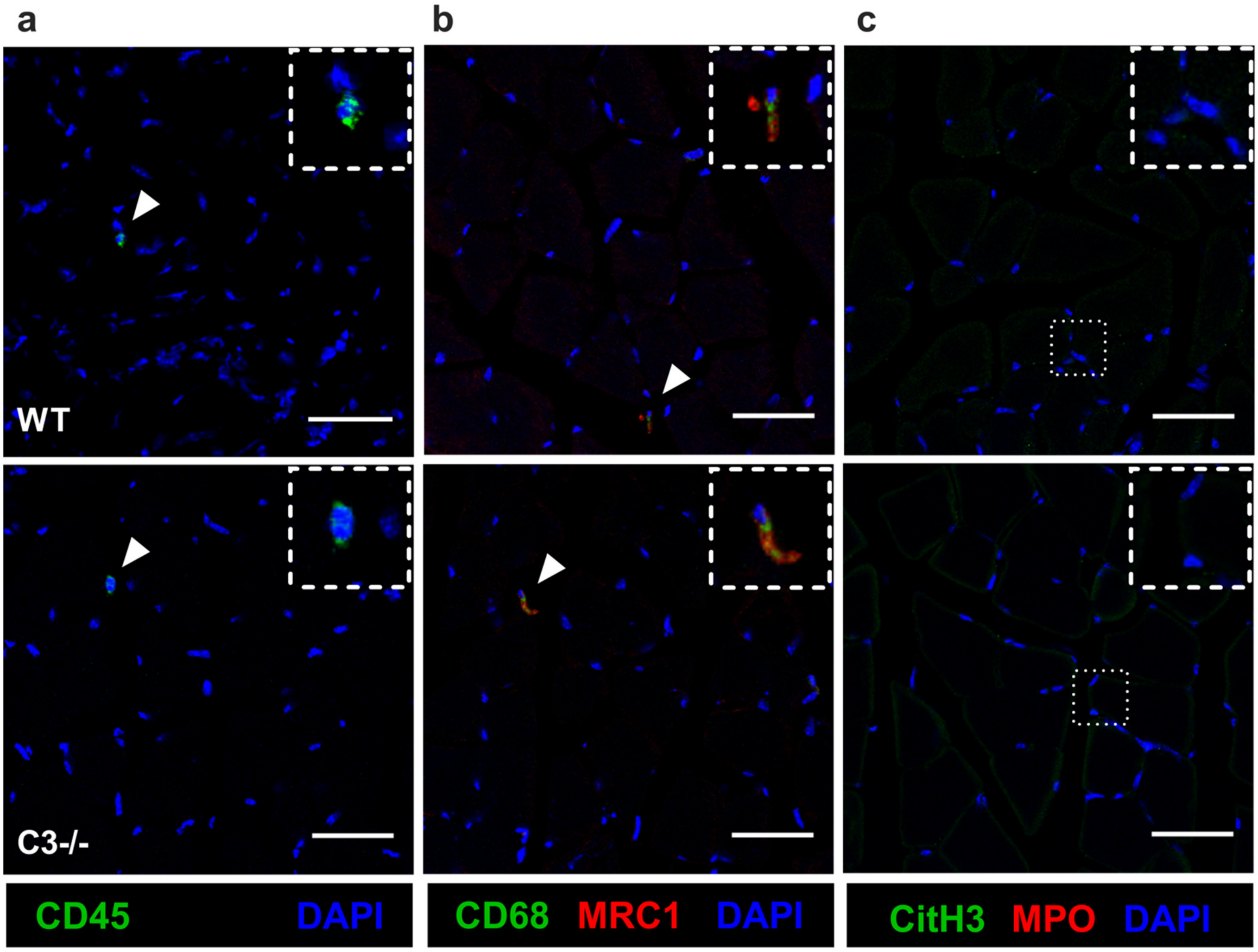

Figure S4. C3−/− and WT show little number of CD45⁺, CD68⁺, and MPO⁺ cells. Representative immunofluorescence pictures of gastrocnemius muscles isolated from sham-operated of WT (upper pictures) and C3−/− mice (lower pictures) 7 days (**a** and **b**) and 24h (**c**) after FAL. (**a**) Cells were labeled with antibodies targeting CD45 (green, pan-leukocyte marker) and with DAPI (blue) to label nuclei. $CD45^+$ cells are indicated by white arrowheads. Scale bars: 50 µm. Detail 30µm × 30µm. (**b**) Cells were labeled with antibodies targeting MRC1 (red, marker for M2-like polarized macrophages), CD68 (green, macrophage marker) and with DAPI (blue) to label the nuclei. $CD68^+$/$MRC1^+$ cells are indicated by white arrowheads. Scale bars: 50 µm. 30µm × 30µm. (**c**) Cells were labeled with antibodies targeting MPO (red, marker for neutrophils), CitH3 (green, citrullinated histone H3) and with DAPI (blue) to label nuclei. No MPO^+ cells or signals for CitH3 can be seen in the representative picture and the cropped detail, referring to the very low amount of MPO^+ cells in tissue of sham operated legs. Colocalized signals for CitH3 and MPO were not found at all. Scale bars: 50µm. Details 30µm × 30µm.
