# Supplementary figures and images for: C3 Deficiency Leads to Increased Angiogenesis and Elevated Pro-Angiogenic Leukocyte Recruitment in Ischemic Muscle Tissue
Source: Int J Mol Sci. 2021 May 28;22(11):5800. doi: 10.3390/ijms22115800 (PMC8198161; doi:10.3390/ijms22115800)

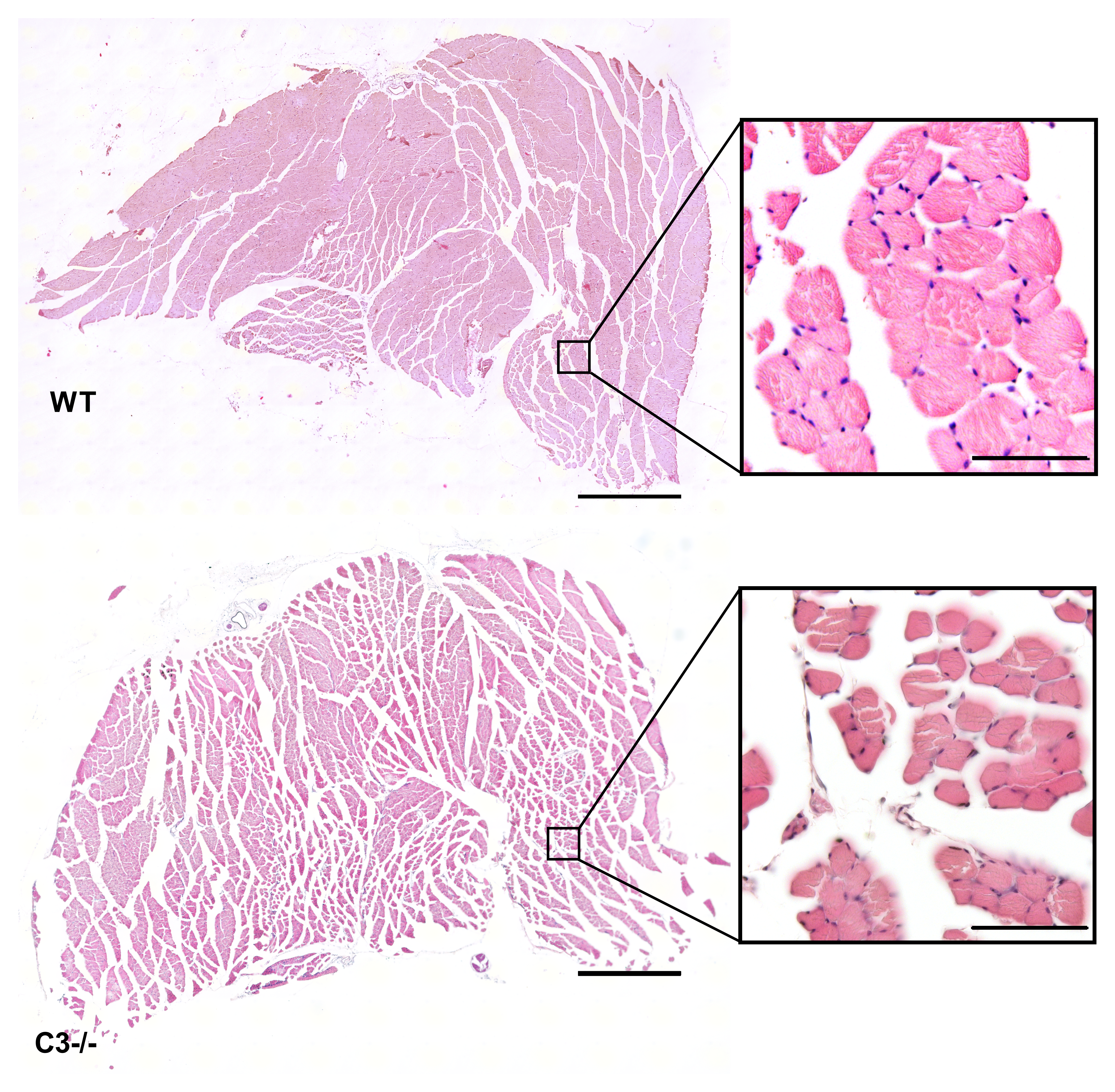

Supplement: Supplementary file 1 [file ijms-22-05800-s001.zip › Supplementary material/Figure S1.tif]

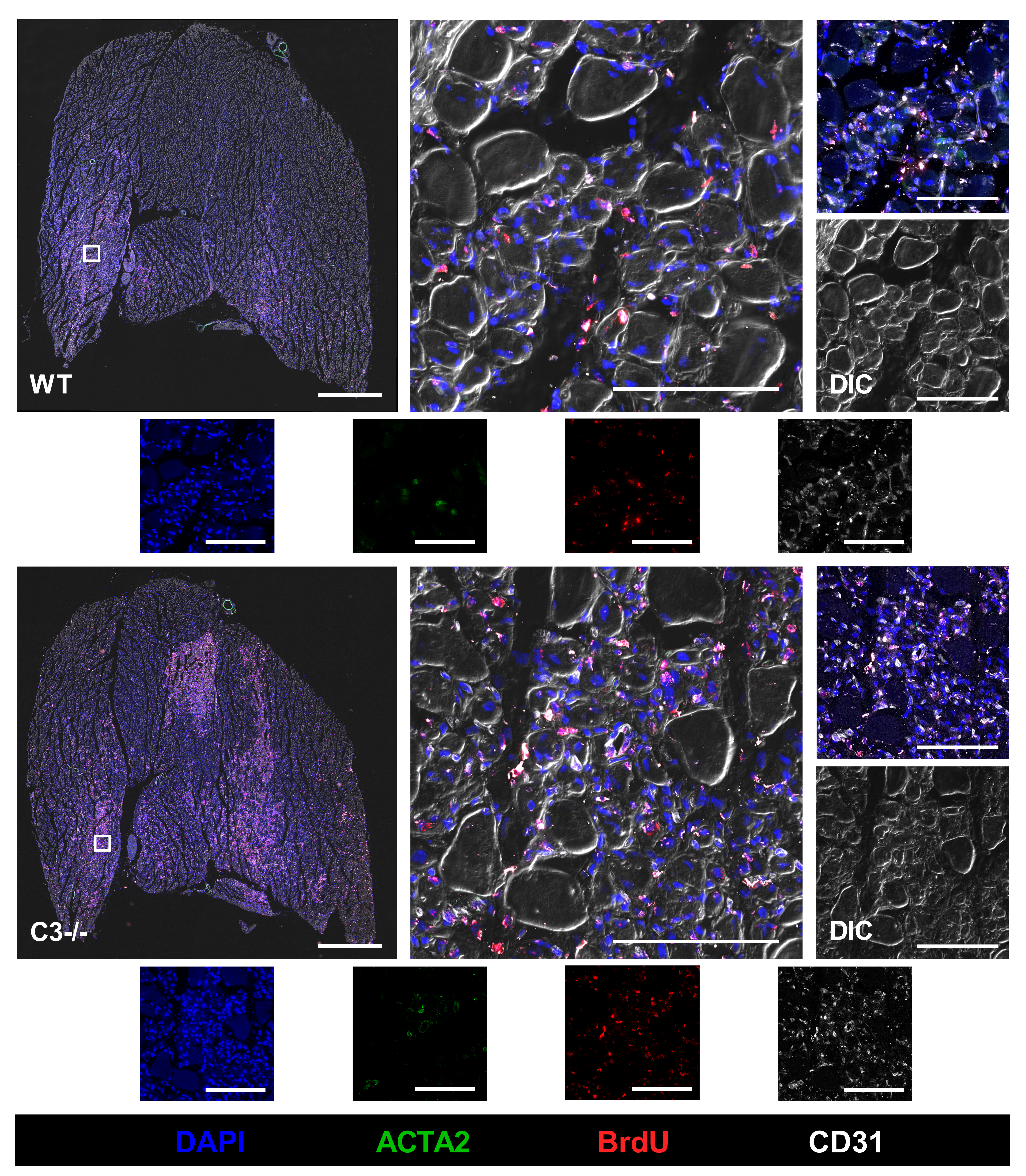

Supplement: Supplementary file 1 [file ijms-22-05800-s001.zip › Supplementary material/Figure S2.tif]

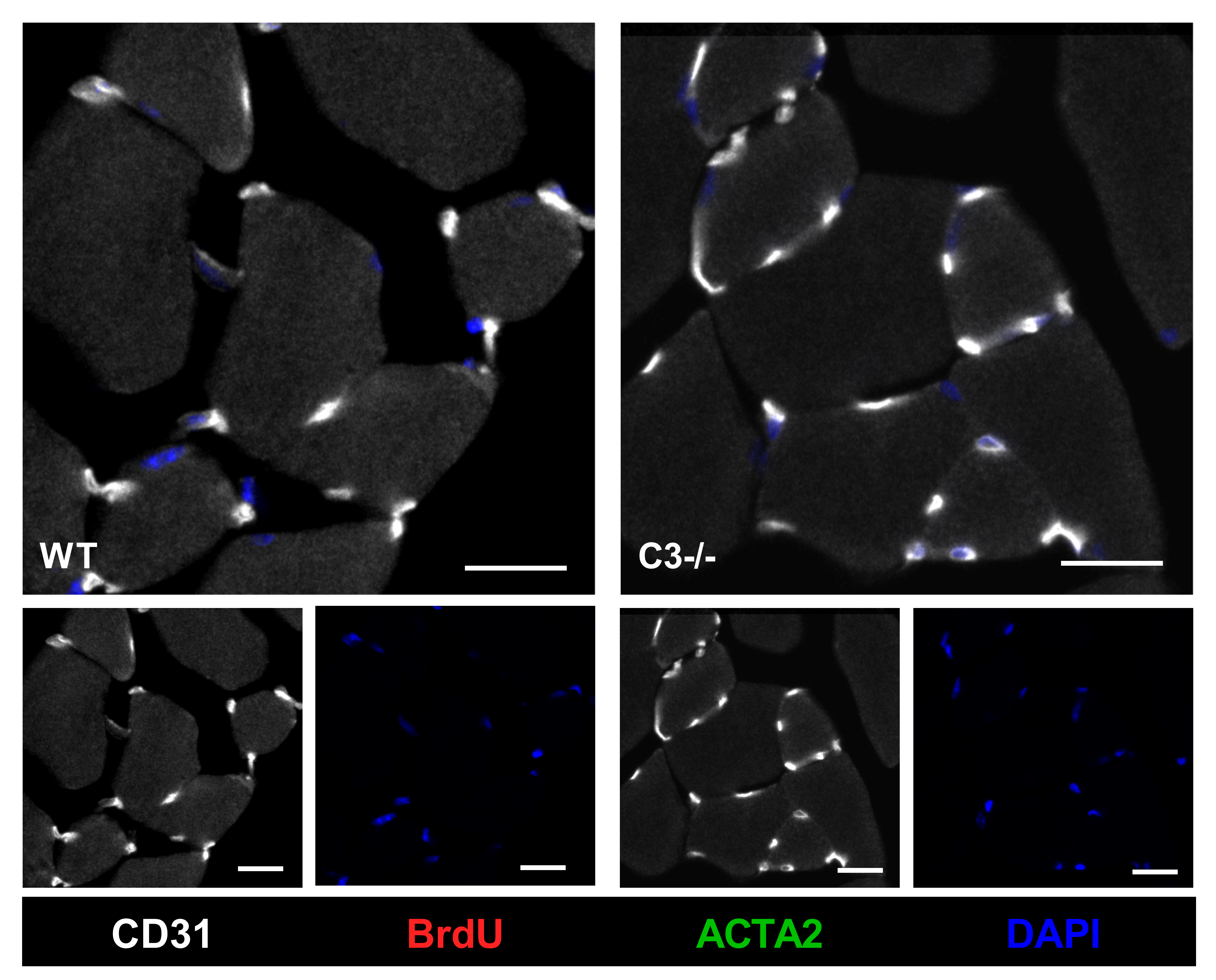

Supplement: Supplementary file 1 [file ijms-22-05800-s001.zip › Supplementary material/Figure S3.tif]

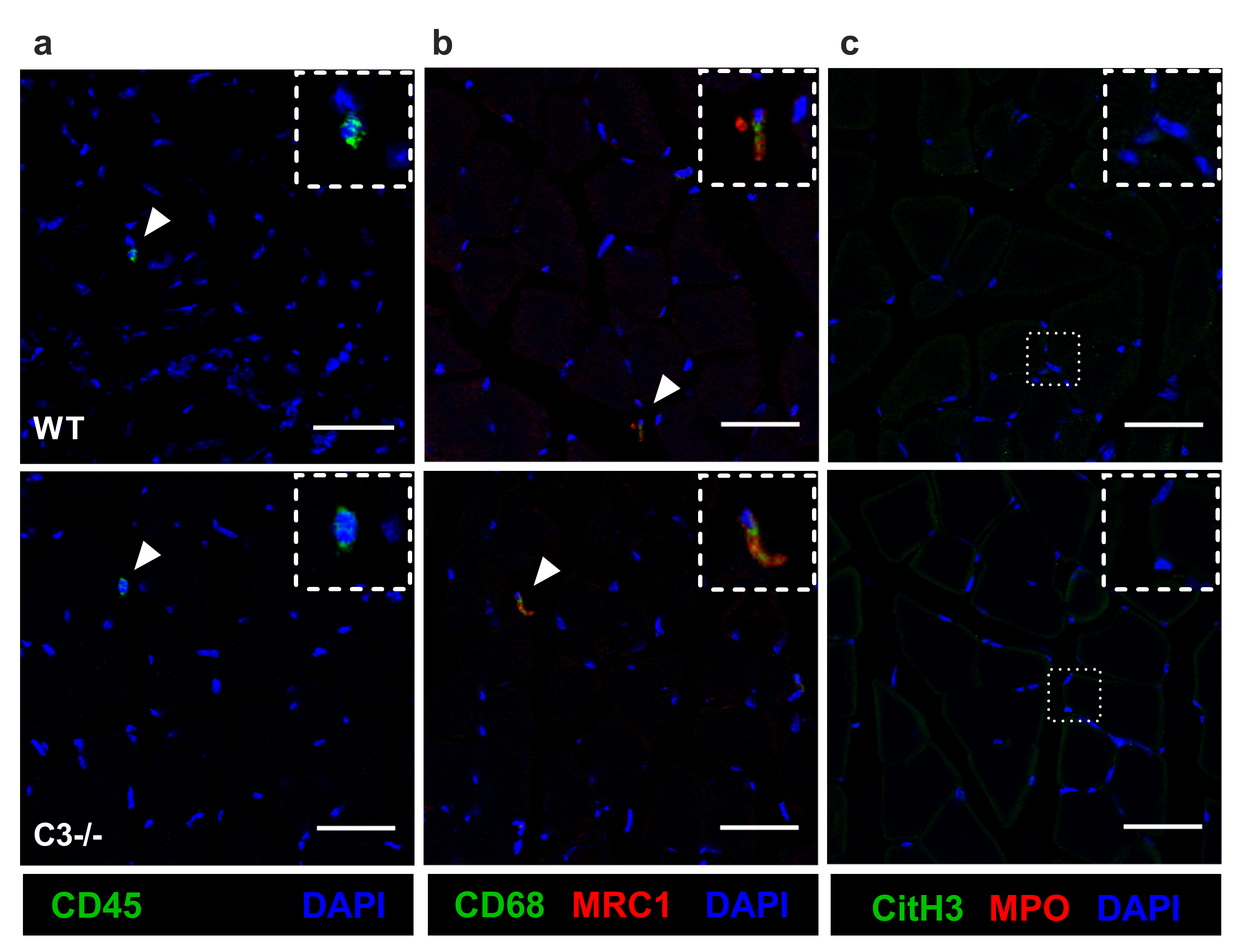

Supplement: Supplementary file 1 [file ijms-22-05800-s001.zip › Supplementary material/Figure S4.tif]
